# Impact of COVID-19 lockdown on glycemic control in patients with type 1 and type 2 diabetes mellitus: a systematic review

**DOI:** 10.1186/s13098-021-00705-9

**Published:** 2021-09-07

**Authors:** Claudia Eberle, Stefanie Stichling

**Affiliations:** Medicine With Specialization in Internal Medicine and General Medicine, Hochschule Fulda–University of Applied Sciences, Leipziger Strasse 123, 36037 Fulda, Germany

**Keywords:** COVID-19, Lockdown, Type 1 diabetes, Type 2 diabetes, Glycemic control, Systematic review

## Abstract

**Background:**

In 2019, a new virus known as severe acute respiratory syndrome coronavirus type 2 (SARS-CoV-2) has emerged. Coronavirus disease 2019 (COVID-19) was classified as a pandemic in a short period of time. In order to reduce the spread of COVID-19, many countries have imposed a lockdown with movement restrictions, social distancing and home confinement, which has affected routine healthcare activities and everyday life. The aim of this systematic review was to examine the impact of the COVID-19 lockdown on glycemic control in patients with type 1 diabetes (T1D) and type 2 diabetes (T2D).

**Methods:**

We systematically identified studies by searching the databases Cochrane Library, MEDLINE via PubMed, Web of Science Core Collection, EMBASE, and CINAHL until April 2021. We included n = 33 observational studies of which n = 25 investigated T1D and n = 8 T2D.

**Results:**

Overall, we analyzed n = 2881 T1D patients and n = 1823 T2D patients. Glycemic values in patients with T1D improved significantly during lockdown. Overall, n = 18 (72%) T1D studies indicated significant improvements in glycemic outcomes. Meta-analysis revealed a mean difference in HbA1c of − 0.05% (95% CI − 0.31 to 0.21) due to lockdown, and in time in range (TIR) of + 3.75% (95% CI 2.56 to 4.92). Lockdown determined a short-term worsening in glycemic values in patients with T2D. Overall, n = 4 (50%) publications observed deteriorations in glycemic control. Meta-analysis demonstrated a mean difference in HbA1c of + 0.14 (95% CI − 0.13 to 0.40) through the lockdown. Moreover, n = 3 (75%) studies reported a not significant deterioration in body weight.

**Conclusions:**

Glycemic values in people with T1D significantly improved during COVID-19 lockdown, which may be associated with positive changes in self-care and digital diabetes management. In contrast, lockdown rather determined a short-term worsening in glycemic parameters in patients with T2D. Further research is required, particularly into the causes and effective T2D management during lockdown.

**Supplementary Information:**

The online version contains supplementary material available at 10.1186/s13098-021-00705-9.

## Background

In 2019, a new virus known as severe acute respiratory syndrome coronavirus type 2 (SARS-CoV-2) has emerged in Wuhan, China and was classified as a pandemic and global public health concern in a short period of time [1]. Since then, 142,238,073 coronavirus disease 2019 (COVID-19) cases were confirmed worldwide and 3,032,124 people died (as of April 21, 2021) [1]. One of the most vulnerable people to this virus are those with chronic diseases such as diabetes mellitus [2–4]. Globally, about 463 million adults (20–79 years) are diagnosed with diabetes [5]. Type 1 diabetes mellitus (T1D) accounts for 5–10% of all diabetes forms and arises mostly in kids and young adults [5]. About 1.1 million children and adolescents are diagnosed with T1D worldwide [5]. T1D is a complex disease dependent on immune system beta-cell demolition prompting total insulin lack [6].

However, type 2 diabetes mellitus (T2D) is the most prevalent type of diabetes with ca. 90–95% [7]. Some risk factors for T2D seem to be rising age, Body Mass Index (BMI) ≥ 25 kg/m^2^, and limited physical activity. T2D arises mainly based on a progressive loss of beta-cell insulin secretion due to insulin resistance [7].

Comorbidities, such as microvascular (eg nephropathy, retinopathy) and macrovascular (eg. cardiovascular disease, stroke, peripheral artery disease) complications are associated with diabetes [6, 8]. Moreover, diabetes leads to a significant increase in COVID-19-related mortality associated with acute respiratory distress syndrome [9].

Optimal glycemic control is important to reduce and prevent diabetic complications and diabetes patients need special care [10]. However, the management of diabetes patients has been changed due to the lockdown imposed to prevent the spread of the pandemic [11]. Governments from different countries have adopted various infection control measures to slow the spread of COVID-19 [11]. Social distancing and quarantine ensure that clinic visits are reduced for fear of infection [12]. Furthermore, the infection control measures might affect physical activity and diet, and there are effects on health care and access to diabetes medication [12]. Due to the contact restrictions and restrictions in the health care area, digital approaches to ensure optimal management of diabetes patients come into focus [12]. Diabetes technology, for example telemedicine, overcomes physical limits and can improve health care access [13].

For example, a study from China found a deterioration in blood glucose control and a high fasting blood glucose during lockdown in T1D patients [14], whereas another study from Spain even found an improvement in glycemic parameters [15]. More and more individual studies from different countries are examining the “lockdown effect” on the management of diabetes patients. In order to generate an overview of the current study situation on this new research topic, we carried out a systematic review. We aimed to investigate the effects of lockdown during the COVID-19 pandemic on glycemic control in patients with type 1 and patients with type 2 diabetes.

## Materials and methods

### Literature search

Publications were systematically identified by searching the databases Cochrane Library, MEDLINE via PubMed, Web of Science Core Collection, EMBASE, and CINAHL for studies published until April 2021.

Search was conducted using the following keywords: (“diabetes mellitus”) AND (“COVID-19” OR “SARS-CoV-2”) AND (“lockdown” OR “isolation” OR “quarantine” OR “glycemic control”). Medical Subject Headings and Embase Subject Headings terms as well as title/abstract terms were searched. For example, our search strategy in PubMed was: ((“diabetes mellitus”)[MeSH Terms])) AND (("SARS-COV-2"[Title/Abstract]) OR ("COVID-19" [MeSH Terms])) AND ((“lockdown”)[Title/Abstract])) OR (“isolation”)[Title/Abstract] OR (“quarantine”)[MeSH Terms] OR (“glycemic control”)[Title/Abstract])).

After searching the five databases, we removed duplicates, screened titles and abstracts for suitability and read the available full texts. In addition, we manually searched reference lists and Google Scholar. Studies were selected by two independent reviewers.

### Inclusion and exclusion criteria

We included peer-reviewed studies published in English and German. We considered observational studies (eg. cohort studies, cross-sectional studies, case–control), retrospective and prospective, that examined the effect of COVID-19-lockdown (infection control measures and their consequences; restrictions, social distancing, quarantine) on glycemic parameters on type 1 and type 2 diabetes patients.

The studies examined a wide variety of outcomes. Glycemic outcomes were heterogeneous. We decided to analyze the most frequently examined outcomes in order to be able to compare the studies as best as possible and to be able to carry out calculations (meta-analysis). Therefore, we analyzed the following most common outcomes: glycated hemoglobin A1c (HbA1c), time in range (TIR) (70–180 mg/dl), and body mass index (BMI)/weight.

In contrast, we have excluded the following aspects: paper which did not specify the type of diabetes, patients with gestational diabetes mellitus and other types of diabetes, duplicates, studies examining other topics and outcomes, the following articles types: poster, abstracts, study protocols, proceeding papers.

### Data extraction

We extracted the following information: year of publication, study region, study design, research objects, patient characteristics, digital care during lockdown, outcomes of interest, key findings (effect of lockdown, statistical information and calculations), study limitations, and hypotheses for underlying reasons regarding the results.

### Data synthesis and analysis

We sorted the publications according to the type of diabetes, outcomes and diabetes technology. The studies were classified according to the type of treatment, since technological diabetes management strategies were used here, such as continuous glucose monitoring (CGM) and flash glucose monitoring (FGM), continuous subcutaneous insulin infusion (CSII), hybrid closed loop system (HCL), sensor-augmented pump (SAP), multiple daily injections (MDI) and telemedicine/teleconsulting (not specified).

In addition, we analyzed the impact of lockdown on outcomes compared to the time before lockdown, and performed a meta-analysis on the most common parameters HbA1c and TIR. We took into account all studies that provided complete information on these outcomes. In the calculations, we considered T1D and T2D studies separately. For determining the changes in HbA1c (%) and TIR (%), we pooled appropriate studies and calculated the difference in means, with 95% confidence intervals (CI) before and during/after lockdown. In studies that noted several measurement times during the lockdown, we selected the last measurement time and not the intermediate results.

## Results

### Overview characteristics of included studies

The literature search yielded n = 767 citations of which n = 662 unique citations were screened based on the defined eligibility criteria (see Additional file 1: Fig. S1). After an additive research of reference lists, we identified n = 33 observational studies of which n = 25 investigated T1D and n = 8 T2D. Characteristics of the included publications are provided in Table 1, whereas a detailed description of the studies is exhibited in Additional file 1.

**Table 1:** Study characteristics and pooled HbA1c and TIR values

	Type 1 diabetes	Type 2 diabetes
Participants (n)	2881	1823
Diabetes technology during lockdown		
CGM (n)	10	–
FGM (n)	5	–
HCL (n)	2	–
SAP (n)	1	–
Different types (n) (CGM, CSII, FGM, insulin pump)	5	–
Telemedicine^a^ (n)	–	5
Not described (n)	2	3
Location		
Italy (n)	11	2
Spain (n)	6	–
Turkey (n)	–	2
India (n)	4	3
Greece (n)	1	1
China (n)	1	–
Israel (n)	1	–
UK (n)	2	–
Saudi Arabia (n)	1	–
Japan (n)	1	–
Outcomes^b^		
Impact lockdown on HbA1c (%) *pooled HbA1c levels*	Mean difference − 0.05 (95% CI − 0.31 to 0.21) *(based on n* = *11 studies)*	Mean difference + 0.14 (95% CI − 0.13 to 0.40) *(based on n* = *8 studies)*
Impact lockdown on time in range (TIR) (%), 70–180 mg/dl *pooled TIR values*	Mean difference + 3.75 (95% CI 2.56 to 4.92) *(based on n* = *18 studies)*	N/A

Italics indicate "pooled" parameters

^a^Telemedical treatment not precisely described

^b^Calculations of the outcomes that have been examined most often

The studies come from the following countries: Italy (n = 13), Spain (n = 6), India (n = 4), Turkey (n = 2), Greece (n = 2), China (n = 1), Israel (n = 1), UK (n = 2), Japan (n = 1), and Saudi Arabia (n = 1). In our analysis, we focused on the most common glycemic parameters [HbA1c, TIR (70–180 mg/dl)], and additionally BMI and weight, as these parameters also occurred frequently.

### Type 1 diabetes

Overall, we analyzed n = 2881 T1D patients. Of n = 25 T1D studies, n = 18 (72%) showed clear, significant improvements in glycemic control [14–31], n = 4 (16%) showed stability (no changes) [32–35] and n = 3 (12%) deteriorations [36–38]. In n = 10 studies, the patients received CGM treatment, in n = 5 studies FGM, in n = 2 studies HCL, in n = 1 study SAP, in n = 5 studies mixed types of technology such as MDI, CSII, CGM, FGM, insulin pump, and in n = 2 papers no digital treatments were described. The age groups of the participants were heterogeneous from children to adolescents, young adults to the elderly, and also varied within the studies.

#### Effect on HbA1c

For determining the changes in HbA1c (%) and TIR (%), we pooled appropriate studies and calculated the difference in means, with 95% confidence intervals (CI) before and during/after lockdown. Meta-analysis showed that, compared to the time before lockdown, HbA1c values declined with a mean difference of − 0.05% (95% CI − 0.31 to 0.21) based on n = 11 eligible studies [15, 21, 23, 25, 27, 28, 30, 32, 36–38]. In n = 8 (73%) studies in which HbA1c values improved significantly (P < 0.05) due to the “lockdown effect”, the improvements ranged between − 0.1% and − 0.3%.

In total, n = 2 (18%) studies reported significant (P = 0.027 [36] and P < 0.05 [38]) deteriorations. Included in the calculations is a study from India [38] that found a large HbA1c deterioration of + 1.2% [8.8 ± 1.3% vs. 10 ± 1.5% (P < 0.05)]. According to Verma et al. [38], this is due to the non availability of insulin/glucostrips during lockdown period. If this study is excluded from the calculations, a mean difference of − 0.16% (95% CI − 0.25 to − 0.07) is obtained (based on n = 10 studies). Moreover, another study by Hosomi et al. [36] reported HbA1c levels became worse: + 0.12% in 2020 vs. − 0.09% 1 year ago (P = 0.027).

#### Effect on time in range (TIR)

Furthermore, we pooled studies to determine the change in TIR (%) (70–180 mg/dl). Based on n = 18 eligible studies, the analysis revealed an effect size of mean difference =  + 3.75% (95% CI 2.56 to 4.92) [15–21, 23–27, 29, 31–33, 35]. Of these studies, n = 15 (83%) indicated a significant improvement (P < 0.05) in TIR and n = 3 (17%) studies found no significant changes (P = 0.168 [32], P = 0.812 [33], P > 0.05 [35]) due to the COVID-19 lockdown.

### Type 2 diabetes

In general, we analyzed n = 1823 T2D patients. Of n = 8 T2D studies, n = 4 (50%) showed clear deteriorations in glycemic control (HbA1c and mean glucose) [39–42], n = 2 (25%) showed stability (no changes) [43, 44] and n = 2 (n = 25%) [45, 46] improvements. In n = 5 of n = 8 T2D studies, the patients received telemedicine care, but telemedicine was not described in detail, and in n = 3 papers no digital treatments were described.

#### Effect on HbA1c

Meta-analysis showed that, compared to the time before COVID-19 lockdown, the lockdown was associated with effect size of mean difference =  + 0.14 (95% CI − 0.13 to 0.40) based on n = 8 suitable papers.

In total, Rastogi et al. and Psoma et al. reported a significant reduction in HbA1c values (6.9% ± 1.3 vs. 6.7% ± 0.9, P = 0.015 [45] and 7.8% (6.9 to 9.4) vs. 7.4% (6.6 to 8.7), P = 0.005 [46]). In contrast, n = 4 studies (50%) observed increased HbA1c values, n = 2 of them significant (P = 0.02 [42] and P = 0.002 [40]) and n = 2 not significant (P = 0.253 [39] and P > 0.05 [41]).

Moreover, the case–control study by Karatas et al. [40] compared a diabetic (n = 85) and a non-diabetic (n = 55) group and found that HbA1c increased significantly in both groups (+ 0.71% ± 1.35 vs. + 0.02% ± 0.19%, P = 0.002).

#### Effect on BMI and weight

Overall, n = 4 studies reported on BMI or weight. One paper (25%) [45] reported a significant improvement in BMI (30.6 kg/m^2^ ± 5.8 vs. 30.3 kg/m^2^ ± 5.6, P = 0.01), whereas n = 3 (75%) studies showed a deterioration in weight (+ 0.3 kg [44]; + 0.54 ± 0.95 kg [40]; + 0.8 kg [39]), but not significant (P > 0.05).

## Discussion

In general, glycemic parameters in patients with T1D significantly improved during COVID-19 lockdown. Overall, n = 18 (72%) T1D studies indicated clear, significant improvements in glycemic outcomes. Meta-analysis demonstrated a mean difference in HbA1c of − 0.05% (95% CI − 0.31 to 0.21) due to “lockdown effect” and in time in range (TIR) of + 3.75% (95% CI 2.56 to 4.92). In contrast, lockdown determined a short-term worsening in glycemic values in patients with T2D. Overall, n = 4 (50%) publications observed clear deteriorations in glycemic control. Meta-analysis demonstrated a mean difference in HbA1c of + 0.14 (95% CI − 0.13 to 0.40) through the lockdown. Furthermore, n = 3 (75%) studies reported a (not significant) deterioration in weight between + 0.3 kg and + 0.95 kg.

COVID-19 lockdown and the associated measures and restrictions led to a change in health care, in people’s everyday lives and also had an impact on diabetes management. Our results demonstrate that the effects of pandemic lockdown on T1D and T2D were very different.

T1D and T2D have a contrasting pathogenesis, which also results in appropriate therapy recommendations with different priorities. Lifestyle changes (eg. diet, exercise) are part of both T1D and T2D management, but the focus of T1D is on insulin therapy [47]. HbA1c could be significantly improved in most studies and this is a promising finding in view of the fact that a good glycemic control reduces the risk of comorbidities and complications as well as progressions of micro- and macrovascular consequences among T1D patients [10]. What is striking about the results is that in almost all cases, T1D patients had digital diabetes management available during the lockdown. Especially digital approaches such as CGM and FGM were often used in T1D therapy.

There are various hypotheses as to why the glycemic values of T1D patients improved as a result of the lockdown. For example, this could be due to more time for self-care and more time to concentrate on T1D management. In the case of children, it may be because the parents who were forced to “stay home” had more time to deal with the disease and family environment may be more attentive in diabetes management [17]. Patients (or their parents) were probably able to balance diet, exercise, and insulin requirements to counteract lockdown consequences such as physical inactivity and the psychosocial impact [15].

Furthermore, the causes can lie in more regular daily lifestyle and a strict daily routine including scheduled meals [19, 21]. In particular, we noticed that digital treatments for T1D patients (using CGM, FGM, HCL and other methods) probably had a positive impact on glycemic control, which the papers also confirm [21, 26, 27, 35]. We have also already examined the effect of various digital diabetes therapy approaches on health outcomes of diabetes patients in other papers, and found that these are generally very promising treatments [48, 49]. However, glucose control may be better likely because of better compliance with medications as well as digital solutions and diet during lockdown was observed in type 1 diabetics despite decreased exercise as the former usually is the main determining factor in blood sugar control among type 1 diabetics.

In contrast, two studies [36, 37] that showed worsening glycemic values hypothesized that this could be due to increased stress and decreased physical activity. One study from India [38] leads back the findings to the non availability of insulin/glucostrips during lockdown period.

Our findings for T2D patients obviously differ from those for T1D patients. Here, the analyzed studies tend to show a deterioration in glycemic values and weight/BMI of the patients due to the lockdown period. In general, the pandemic lockdown can have effects on the lifestyle that could affect health and diabetes management, for example changes in the diet (eg. consumption of calorie-dense foods), less physical activity, more screen time and weight gain [50]. Observations of lifestyle changes during lockdown in T2D patients of Ghos et al. [51] and Ruiz-Roso et al. [52] showed an increase in vegetable, sugary food and snack consumption. They found an association between levels of foods cravings and snack consumption as well as high percentage of physical inactivity before the COVID-19 lockdown which intensified during the home confinement. Other causes might be insufficient sleep, lack of dietary limitation, increased sitting time, increased socioeconomic difficulties alter healthy nutrition [40, 41], the inability to visit hospitals or pharmacies as well as increased anxiety and stress [39]. Besides, there have been other investigations on natural disasters and their effects on diabetes management [53, 54]. Observations of worsening of glycemic outcomes were made during other stressful situations like earthquakes, hurricane and war.

Furthermore, the measures and restrictions that the governments have issued in the context of the COVID-19 pandemic differ from country to country [11]. Some are more and some are less strict, which might lead to different effects on the health system and peoples lifes. In addition, the individual studies have different investigation periods. Some papers observed the impact of lockdown *during* lockdown and some *after* lockdown (compared to pre-pandemic times). However, it must also be considered that, as a reaction to the respective current regional infection process, the lockdowns took place at different periods of time from country to country and also they also differed in number, length and severity. Many of the publications identified come from Italy and Spain. These two European countries had a particularly strict lockdown with severe restrictions for people, e.g. only leaving the apartment for a valid reason (curfew during the day).

Furthermore, it would be interesting and important to investigate pregnant patients with diabetes on this topic, but currently we could only identify one suitable study for this cohort that showed lower diabetes control in patients with gestational diabetes [55].

## Limitations

We only included English and German literature. Furthermore, COVID-19 is a rapidly growing issue. The following must be taken into account when considering our analysis: within the last year, COVID-19-related studies were often published quickly and in a simplified form in order to rapidly generate evidence and derive clinical recommendations, so that formal criteria and detailed information (for example on the methodological approach and with regard to the findings) were deferred. The methodology of the studies and the results as well as statistical information have therefore in some cases not been described in detail by the authors.

In addition, we could only identify a few studies with regard to T2D patients. More research is needed here. Overall, the outcomes were partly heterogeneous.

As already mentioned, the local lockdown measures and restrictions are heterogeneous and we could only evaluate the current data situation. Furthermore, the sample sizes were rather small and further investigations on this new topic, in particular on T2D and diabetes in pregnancy, are necessary.

## Conclusions

To our knowledge, this review presents the first overview of the impact of the COVID-19 lockdown on glycemic values of T1D and T2D patients. Glycemic parameters in patients with T1D significantly improved during COVID-19 lockdown. In contrast, lockdown determined a short-term worsening in glycemic values in patients with T2D.

In particular, the causes of the analyzed developments must be further researched. A detailed analysis of factors capable of affecting patients’ glycemic values, such as lifestyle changes, is essential. In addition, political preparation for pandemics is important, especially in countries where glycemic parameters have deteriorated due to inadequate health care (e.g. lack of insulin). Further and detailed analyzes of (other) outcomes are necessary. Analyzes are also essential at a later point in time, when more patient data are published and available. Larger studies from different locations are necessary to identify the actual impact of lockdown in people with T1D and T2D on a broader scale.

## Supplementary Information

**Additional file 1: Figure S1.** PRISMA flow chart. **Table S1.** Overview of Type 1 Diabetes studies. **Table S2.** Overview of Type 2 Diabetes studies.

## Data Availability

All data generated and analyzed during are included within the article and its Additional files.

## Abbreviations

BMI: Body mass index
CGM: Continous glucose monitoring
CSII: Continuous subcutaneous insulin infusion
COVID-19: Coronavirus disease 2019
FBG: Fasting blood glucose
FGM: Flash glucose monitoring
HbA1c: Glycated hemoglobin A1c
HCL: Hybrid closed loop system
MDI: Multiple daily injections
SAP: Sensor-augmented pump
SARS-CoV-2: Severe acute respiratory syndrome coronavirus type 2
T1D: Type 1 diabetes
T2D: Type 2 diabetes
TIR: Time in range
