# Supplementary material for: Impact of COVID-19 lockdown on glycemic control in patients with type 1 and type 2 diabetes mellitus: a systematic review
Source: Diabetol Metab Syndr. 2021 Sep 7;13:95. doi: 10.1186/s13098-021-00705-9 (PMC8423337; doi:10.1186/s13098-021-00705-9)
Supplement: Supplementary file 1 — Additional file 1: Figure S1. PRISMA flow chart. Table S1. Overview of Type 1 Diabetes studies. Table S2. Overview of Type 2 Diabetes studies. [file 13098_2021_705_MOESM1_ESM.docx]

**Figure S1: PRISMA flow chart.**

Records excluded based on
title and abstract (n=624)

Other COVID-19 topics instead of “lockdown effect” (n=366)

Article type (poster, study protocols, proceedings papers etc.) (n=158)

Wrong population (other type of diabetes, mixed population etc.) (n=48)

Other Outcomes (n=30)

Records identified through database (PubMed, CINAHL, EMBASE, Cochrane Library, Web of Science Core Collection) searching

(n=767)

April 10, 2021

Identification

Citations screened without duplicates
(n=662)

Screening

Studies with full-text assessed for eligibility
(n=38)

Publications excluded (n=7):

No access to full-text (n=1)
Publication retracted (n=1)
Diabetes type not defined (n=1)
Other outcomes (n=4)

Eligibility

After assessing
studies with full-text
(n=31)

Manual research
(n=2)

Finally included in
this review
(n=33)

Type 1 diabetes (n=25)
Type 2 diabetes (n=8)

Tdd

Included

**Table S1: Overview of Type 1 Diabetes studies**

| Author, year, location | Study design | Research object | Patients, n | Digital care during lockdown | Outcomes | Hypotheses for underlying reasons | Overall effect on glycemic control |
| --- | --- | --- | --- | --- | --- | --- | --- |
| (Moreno-Domínguez et al. 2020)  Spain | Observational, retrospective | Factors related to improvement of glycemic control among adults with type 1 diabetes during lockdown due to COVID-19 | N=138 | CGM | Improvement in **mean glucose** P<0.001  Before Lockdown: 155.5 ± 28.6 mg/dL During Lockdown: 149.0 ± 26.8 mg/dL  Improvement **TIR** (70-180 mg/dL) P<0.001 Before Lockdown: 63.8 ± 15.2 %  During Lockdown: 66.7 ± 15.2 % | More time for self-management, regularity of daily schedules, type of food and physical activity | Improvement |
| (Di Dalmazi et al. 2020)  Italy | Cohort | Comparison of the effects of lockdown due to COVID-19 on glucose patterns among children, adolescents, and adults with type 1 diabetes | N=130  (Children n=30  Teenager n=24  Adults n=76) | CGM | CGM metrics were comparable in teenagers before and during lockdown  After lockdown, adults improved significantly  TIR (**TIR**) 70–180 mg/dL (P<0.001)  and **mean glucose**: 164 (145–191) vs 160 (141–175) (P<0.001) | In adults, age, physical  activity, and perceived stress may be relevant contributing  factors  Lockdown effects are and age-specific, being  improved in children and unchanged in teenagers  Family environment and  its pace of life would seem to be more attentive in diabetes management | Improvement |
| (Pla et al. 2020)  Spain | Cohort | Impact of COVID-19 Lockdown on Glycemic Control in Adults with Type 1 Diabetes Mellitus | N=50 | CGM | Reduction of **mean glucose** values: 160.26 ± 22.55 mg/dL vs 150 ± 20.96 mg/dL, P=.0009; estimated **HbA1c**: 7.21 ± 0.78% vs 6.83 ± 0.71%, P=.0005; **TIR** showed an improvement: 57.46 ± 11.85% vs 65.76 ± 12.09%, P<.0001 | Patients were able to balance food intake, home exercise, and insulin requirements  to counteract the consequences of physical  inactivity as well as the psychosocial impact | Improvement |
| (Tinti et al. 2021)  Italy | Cohort | Impact of lockdown during COVID‑19 emergency on glucose metrics of children and adolescents with type 1 diabetes in Piedmont, Italy | N=66 | CGM | **Mean glucose** 168 ± 61 mg/dL (9.3 ± 3.4 mmol/L) vs lockdown 165 ± 58 mg/dL (9.2 ± 3.2 mmol/L); P<0.053,  **TIR** increased from 59.7 ± 13% to 62.5 ± 14% P=0.001 | Less stress related to school and diabetes management | Improvement |
| (Mesa et al. 2020)  Spain | Observational | The impact of strict COVID-19 lockdown in Spain  on glycemic profiles in patients with type 1 Diabetes prone to hypoglycemia using standalone  continuous glucose monitoring | N=92 | CGM | **TIR** 70–180 mg/dL (59.3 ± 16.2 vs 62.6 ± 15.2%) improved significantly P<0.05 | Strict daily routine | Improvement |
| (Predieri et al. 2020)  Italy | Oberservational | Glycemic Control Improvement in  Italian Children and Adolescents With Type 1 Diabetes Followed Through Telemedicine During Lockdown Due to the COVID-19 Pandemic | N=62 | CGM | **Glucose standard deviation** (P<0.0001) improved  Median **TIR** increased from  60.5% to 63.5% (P=0.008) | More regular lifestyle, including scheduled mealtimes without higher  food intake and more frequent snacking, and more time to  concentrate on T1D care by parents who were forced to “stay home” | Improvement |
| (Capaldo et al. 2020)  Italy | Cohort | Blood Glucose Control During Lockdown for COVID-19: CGM Metrics in Italian Adults With Type 1 Diabetes | N=207 | CGM | **Mean glucose** (mg/dL) 9.6 ± 2.0 vs 9.5 ± 1.9 P=0.165  Estimated **HbA1c** (%) 7.7 ± 1.3 vs 7.6 ± 1.1 P=0.098  **TIR** (%) 55.6 ± 17.6 vs 58.2 ± 18.1 P=0.002 | More regular lifestyle, including reproducible  mealtimes and moretime for self-care; teleconsulting | Improvement |
| (Brener et al. 2020)  Israel | Observational | Lessons learned from the continuous glucose monitoring metrics in pediatric patients with type 1 diabetes under COVID‑19 lockdown | N=102 | CGM | **TIR** was 60.9 ± 14.3% before lockdown, with no significant change during lockdown (delta-TIR was 0.9 ± 7.9%) | Good adaption to changing reality, use of CGM | Stable |
| (Wu et al. 2021)  China | Oberservational | Glycemic control in children and teenagers with type 1 diabetes around lockdown for COVID-19 | N=43 | CGM | **TIR** similar before, during, after lockdown | Individuals reduced their physical activity, received longer sleep duration and spent more time on diabetes management | Stable |
| (Christoforidis et al. 2020)  Greece | Observational | Coronavirus lockdown effect on type 1 diabetes management οn  children wearing insulin pump equipped with continuous glucose  monitoring system | N=34 | CGM | **Mean glucose** (mg/dl) 181.41 ± 5.02 vs 184.47 ± 5.15; P=0.397  **TIR** (70-180mg/dl) (%) 60.71 ± 2.27 vs 60.50 ± 2.53; P=0.812 | - | Stable |
| (Bonora et al. 2020)  Italy | Cohort | Glycaemic Control Among People with Type 1 Diabetes During Lockdown for the SARS-CoV-2  Outbreak in Italy | N=33 | FGM | **Mean glucose** declined from  177 ± 45 mg/dl (week before lockdown) to  160 ± 40 mg/dl (lockdown; P=0.005)  **TIR** increased from 54.4 to 65.2%  (P=0.010) | More time to concentrate on diabetes management; more regular lifestyle, including the  timing and composition of meals; less workplace stress | Improvement |
| (Dover et al. 2021)  UK | Observational | Assessment of the effect of the COVID-19 lockdown on glycaemic  control in people with type 1 diabetes using flash glucose  monitoring | N=572 | FGM | **TIR** increased between March and May 2020 [median 53 (41–64)% vs 56 (45–68)%; P<0.001]  Standard deviation of **glucose** (P <0.001)  Estimated **HbA1c** (P <0.001) (%): 7.6 (7.0 to 8.4) vs 7.4 (6.7 to 8.0) | - | Improvement |
| (Caruso et al. 2020)  Italy | Observational | Reduction of hypoglycaemia, lifestyle modifications and  psychological distress during lockdown following  SARS‐CoV‐2 outbreak in type 1 diabetes | N=48 | FGM | No changes were found in TIR, time above range, mean sensor glucose (P>0.05)  **TIR**: 60.8 ± 14.1 vs 60.8 ± 17.4 (lockdown), P>0.9 | Lockdown‐related prolonged exposure to unhealthy  lifestyle but independently of psychological distress | (Partial) improvements |
| (Fernández et al. 2020)  Spain | Observational | Impact of covid-19 lockdown on glycemic control in patients with type 1 diabetes | N=307 | FGM | **Mean glucose** decreased from 166.89±29.4 mg/dL to 158.0±29.0; P<0.001  estimated **HbA1c** declined from 7.4±1.0 to 7.1±1.0% (54±10.9 vs 57±10.9 mmol/mol; P<0.001)  **TIR** increased from 57.8±15.8 to 62.46±16.1%; P<0.001 | greater stability in schedules, healthier meals, and more time to make treatment adjustments | Improvement |
| (Cotovad-Bellas et al. 2020)  Spain | Observational | The challenge of diabetes home control in COVID-19 times: Proof is in the pudding | N=44 | FGM | **Mean glucose** (mg/dL) 165 ± 33 vs 161 ± 40 P=0.240  Estimated **HbA1c** (%) 7.2 ± 0,8 vs 7.1 ± 0,9 P=0.204  **TIR** (%) 58.0 ± 18.1 vs 60.6 ± 20.0, P=0.168 | More time for doing sport and healthy cooking | Stable |
| (Tornese et al. 2020)  Italy | Cohort | Glycemic control in type 1 diabetes mellitus during COVID-19 quarantine and the role of in-home physical activity | N=13 | HCL | **Mean glucose** (mg/dL): 155 (152-168) vs 153 (149-159) (lockdown); P>0.05  **TIR** (70-180 mg/dl) (%): 68 (60-71) vs 72 (68-76) (lockdown); P<0.05 | Improvement in those who continued physical activity during the quarantine | Improvement |
| (Longo et al. 2020)  Italy | Cohort | Glycemic control in people with type 1 diabetes  using a hybrid closed loop system and followed  by telemedicine during the COVID-19 pandemic  in Italy | N=30 | HCL | **TIR** (70–180 mg/dL), %: 68.5 (65, 74) vs 73.5 (66, 81)  **Mean glucose**, mg/dL 155 (149, 164) vs 153 (145, 163); P=0.004) | Incisive change of lifestyle habits imposed by the quarantine in young  people (age range 21–28 years); use of telemedicine | Improvement |
| (Viñals et al. 2021)  Spain | Oberservational | Management of glucose profile throughout strict COVID‑19 lockdown by patients with type 1 diabetes prone to hypoglycaemia using sensor‑augmented pump | N=59 | Sensor-augmented pump (SAP) | **TIR** 70–180 mg/dL (67.6 ± 11.8 vs. 69.8 ± 12.0%); P<0.05  estimated **HbA1c** (6.94 ± 0.8 vs. 6.75 ± 0.7%); P<0.05 | Strict daily routine at home and using SAP | Improvement |
| (Cognigni et al. 2021)  Italy | Cohort | HbA1c and BMI after lockdown for COVID-19 in children and adolescents with type 1 diabetes mellitus | N=50 | CSII, CGM, FGM | Improvement **HbA1c** P=0.04  Before Lockdown: 7.6 (7.0-8.2) % Post Lockdown: 7.4 (7.0-8.1) %  **BMI** increased P=0.81 Before: 0.27 SDS (-0.27-1.18) Post: 0.35 SDS (-0.23-1.29) | More regular timetable during the day and  the continual presence of parents at home | Improvement |
| (Aragona et al. 2020)  Italy | Cohort | Type 1 diabetes and COVID-19: the “Lockdown effect” | N=63 | CGM, FGM | **TIR** increased 58% vs 62%; P<0.05  **Mean glucose** 165 vs. 158 mg/dl, P=0.040 | More regular daily life activities and reduces work-related distress. | Improvement |
| (Marigliano and Maffeis 2021)  Italy | Cohort | Glycemic control of children and adolescents with type 1 diabetes  improved after COVID‑19 lockdown in Italy | N=233 | MDI, CSII, CGM | Higher **BMI** 20.9 ± 3.7 kg/m2 vs. 21.5 ± 4.6 kg/  m2, P<0.05  Lower **HbA1c** 7.82 ± 0.84 vs. 7.44 ± 0.83, P<0.001  Lower **mean glucose** (mg/dl) 178.6 ± 31.2 vs. 169.1 ± 28.6, P<0.001  Higher %**TIR** 52.6 ± 15.2 vs. 58.0 ± 15.1, P<0.001 | Better take care of glucose control, maintaining  a more regular and suitable schedule | Improvement |
| (Prabhu Navis et al. 2021)  England | Cohort | Impact of COVID‑19 lockdown on flash and real‑time glucose sensor  users with type 1 diabetes in England | N=269 | MDI, insulin pump | **%TIR** 3.9–10 mM significantly increased during period 1 (59.6 ± 18.2 vs. 57.5 ± 17.2%, P=0.002) and period 2 (59.3 ± 18.3  vs. 57.5 ± 17.2%, P=0.035) | Positive changes in self-management strategies | Improvement |
| (Hosomi et al. 2020)  Japan | Cohort | Effect of COVID-19 pandemic on the lifestyle and glycemic control in patients with type 1 diabetes: A  retrospective cohort study | N=34 | MDI, CSII | **HbA1c** was become worse: 0.12  [0.33] % in this year vs. -0.09 [0.39] % in 1 years ago, P=0.027) | Reducing exercise and  increasing stress | Deterioration |
| (Al Agha et al. 2021)  Saudi Arabia | Cross-sectional | Impact of COVID-19 lockdown on glycemic control in  children and adolescents | N=150 | Not specified | Significant increase in patients’ **weight** (P=0.001) and **body mass index** (P=0.001),  **HbA1c (%), P=0.765**  Before lockdown 7.45 ± 1.67  During lockdown 7.40 ± 1.54  **Blood glucose readings** (P=0.007)  Before lockdown 182.09 ± 76.68 mg/dL  During lockdown 200.45 ± 79.97 | decreased physical activity and an unhealthy diet, distortion in people’s daily routines | Deterioration |
| (Verma et al. 2020)  India | Cross-sectional | Impact of lockdown in COVID 19 on glycemic control in patients with  type 1 Diabetes Mellitus | N=52 | Not specified | **Mean blood glucose** 212.3 ± 57.9 mg/dl vs  276.9 ± 64.7 mg/dl  Mean **HbA1c** value 8.8 ± 1.3% vs 10 ± 1.5% (P<0.05). | Non availability of insulin /  glucostrips during lockdown period | Deterioration |

BMI: Body mass index; CGM: continous glucose monitoring; CSII: continuous subcutaneous insulin infusion; DM: Diabetes mellitus; COVID-19: coronavirus disease 2019 FBG: fasting blood glucose; FGM: flash glucose monitoring; HbA1c: glycated hemoglobin A1c; HCL: hybrid closed loop system; MDI: multiple daily injections; SAP: Sensor-augmented pump; SARS-CoV-2: severe acute respiratory syndrome coronavirus type 2; T1D: type 1 diabetes; T2D: type 2 diabetes; TIR: time in range

**Table S2: Overview of Type 2 Diabetes studies**

| Author, year, location | Study design | Research object | Patients, n | Digital care during lockdown | Outcomes | Hypotheses for underlying reasons | Overall effect on glycemic control |
| --- | --- | --- | --- | --- | --- | --- | --- |
| (Rastogi et al. 2020)  India | Cohort, prospective | Improved glycemic control amongst people with long-standing  diabetes during COVID-19 lockdown | N=422 | Telemedicine, self monitoring blood glucose | decrease in **HbA1c** from 7.8% (6.9 to 9.4) prior lockdown to 7.4% (6.6 to 8.7) during lockdown; P=0.005 | Awareness of glycemic goals, access to self-monitoring of blood glucose, and ability to cope with restrictions of lockdown | Improvement |
| (Psoma et al. 2020)  Greece | Observational | Effect of COVID-19-associated lockdown on the metabolic control of patients with type 2 diabetes | N=380 | Not specified | **BMI** (Kg/m2) 30.6 ± 5.8 vs 30.3 ± 5.6, P=0.01  **Glucose** (mg/dl) 127 ± 42 vs 123 ± 35; P>0.05  **HbA1c** (%) 6.9 ± 1.3 vs 6.7 ± 0.9, P=0.015 | Better eating behavior in home-office (home-prepared meals which are better than take-aways at the office), chance for more exercise | Improvement |
| (Sankar et al. 2020)  India | Observational | Effects of COVID-19 lockdown on type 2 diabetes, lifestyle and  psychosocial health: A hospital-based cross-sectional survey from  South India | N=110 | Telemedicine used by 7.3% | **HbA1c** before (8.2 ± 1.3%) and after (8.12 ± 1.6%) lockdown, P>0.05  **Weight** after lockdown higher (71.8 ± 13.6 kg) vs (71.5 ± 14.8 kg), P>0.05 | Lockdown did not cause a major disruption in glycemic control, lifestyle and psychosocial health | Stable |
| (Falcetta et al. 2021)  Italy | Observational | Impact of COVID-19 lockdown on glucose control of elderly people with type 2 diabetes in Italy | N=304 | Teleconsultation | **HbA1c**, % (7.1±0.9 vs 7.1±0.9; P=0.600)  Worsening of HbA1c occurred more frequently in older patients (32.2% in >80  years vs 21.3% in 61-80 years vs 9.3% in < 60 years; P=0.05) | Limited impact of the lockdown on metabolic control | Stable |
| (Khare and Jindal 2020)  India | Observational | Observational study on effect of lock down due to COVID 19 on HBA1clevels in patients with diabetes: Experience from Central India | N=307 | Telemedicine, but only a few patients (not described in detail) | 7.92% mean pre-lockdown **HbA1c** vs 8.43% after release of lockdown, P=0.02 | Type of diet due to being at home, lack of exercise and psychological stress | Deterioration |
| (Biancalana et al. 2020)  Italy | Observational, prospective | Short‑term impact of COVID‑19 lockdown on metabolic control  of patients with well‑controlled type 2 diabetes | N=114 | Telemedicine | **HbA1c** (%) mean pre-lockdown: 6.7 ± 0.7 vs post-lockdown 6.8 ± 0.9, P>0.05 | reduced physical activity and more sedentary lifestyle | Deterioration |
| (Karatas et al. 2021)  Turkey | Prospective Case-control | Impact of lockdown COVID-19 on metabolic control in type 2 diabetes mellitus and healthy people | N=85 diabetic and n=55 non-diabetic | Not specified | **BMI** increased both in non-diabetic (0.54 ± 0.95 kg) and diabetic groups (1.91 ± 5.48 kg) (P>0.05)  Increase in **HbA1c** was more in diabetic than in non-diabetic groups (0.71 ± 1.35 vs. 0.02 ± 0.19%, P=0.002)  **Glucose**, LDL-C, and TG increased in diabetic (39.69 ± 74.69, 7.60 ± 34.33, and 58.21 ± 133.54 mg/dl, P<0.05) whereas non significantly decreased in non-diabetic group (−0.51 ± 4.40, −3.52 ± 14.53, and −6.47 ±41.77 mg/dl) | Insufficient sleep, post-dinner snacking, lack of dietary limitation, eating in reaction to stress, and diminished physical activity  Increased socioeconomic difficulties alter healthy nutrition, increased sitting time | Deterioration |
| (Önmez et al. 2020)  Turkey | Observational | The effect of COVID-19 lockdown on glycemic control in patients with  type 2 diabetes mellitus in Turkey | N=101 | Not specified | **HbA1c** rose from 7.67 ± 1.76 to 8.11 ± 2.48, P=0.253  **Weight** 84.7 ± 16.4 kg rising to 85.5 ± 16.8 kg, P=0.781 | restriction of activity and dietary changes,  inability to visit hospitals or pharmacies,  increasing anxiety and stress | Deterioration |

BMI: Body mass index; CGM: continous glucose monitoring; CSII: continuous subcutaneous insulin infusion; DM: Diabetes mellitus; COVID-19: coronavirus disease 2019 FBG: fasting blood glucose; FGM: flash glucose monitoring; HbA1c: glycated hemoglobin A1c; HCL: hybrid closed loop system; SARS-CoV-2: severe acute respiratory syndrome coronavirus type 2; T1D: type 1 diabetes; T2D: type 2 diabetes; TIR: time in range

References

1. World Health Organization. WHO Coronavirus (COVID-19) Dashboard | WHO Coronavirus Disease (COVID-19) Dashboard. 05.03.2021. https://covid19.who.int/. Accessed 5 Mar 2021.

2. Verity R, Okell LC, Dorigatti I, Winskill P, Whittaker C, Imai N, et al. Estimates of the severity of coronavirus disease 2019: a model-based analysis. The Lancet Infectious Diseases. 2020;20:669–77. doi:10.1016/S1473-3099(20)30243-7.

3. Kleinwechter H, Laubner K. Coronavirus disease 2019 (COVID-19) and pregnancy: Overview and report of the first German case with COVID-19 and gestational diabetes. Diabetologe. 2020;16:242–6. doi:10.1007/s11428-020-00611-0.

4. Bajgain KT, Badal S, Bajgain BB, Santana MJ. Prevalence of comorbidities among individuals with COVID-19: A rapid review of current literature. American Journal of Infection Control 2020. doi:10.1016/j.ajic.2020.06.213.

5. International Diabetes Federation. IDF Diabetes Atlas 9th edition 2019. 2020. https://www.diabetesatlas.org/en/resources/.

6. American Diabetes Association. 2. Classification and Diagnosis of Diabetes: Standards of Medical Care in Diabetes-2020. Diabetes Care. 2020;43:S14-S31. doi:10.2337/dc20-S002.

7. American Diabetes Association. 2. Classification and Diagnosis of Diabetes: Standards of Medical Care in Diabetes-2019. Diabetes Care. 2019;42:S13-S28. doi:10.2337/dc19-S002.

8. Viigimaa M, Sachinidis A, Toumpourleka M, Koutsampasopoulos K, Alliksoo S, Titma T. Macrovascular Complications of Type 2 Diabetes Mellitus. Curr Vasc Pharmacol. 2020;18:110–6. doi:10.2174/1570161117666190405165151.

9. Gupta R, Ghosh A, Singh AK, Misra A. Clinical considerations for patients with diabetes in times of COVID-19 epidemic. Diabetes Metab Syndr. 2020;14:211–2. doi:10.1016/j.dsx.2020.03.002.

10. Kleinwechter H. Diabetes and pregnancy—update 2020. Diabetologe. 2020;16:470–7. doi:10.1007/s11428-020-00629-4.

11. Desvars-Larrive A, Dervic E, Haug N, Niederkrotenthaler T, Chen J, Di Natale A, et al. A structured open dataset of government interventions in response to COVID-19. Sci Data. 2020;7:285. doi:10.1038/s41597-020-00609-9.

12. Scott ES, Jenkins AJ, Fulcher GR. Challenges of diabetes management during the COVID-19 pandemic. Med J Aust. 2020;213:56-57.e1. doi:10.5694/mja2.50665.

13. World Health Organization. Telemedicine: Opportunities and Developments in Member States. Report on the Second Global Survey on eHealth 2009. Geneva: World Health Organization; 2010.

14. Tornese G, Ceconi V, Monasta L, Carletti C, Faleschini E, Barbi E. Glycemic Control in Type 1 Diabetes Mellitus During COVID-19 Quarantine and the Role of In-Home Physical Activity. Diabetes Technol Ther. 2020;22:462–7. doi:10.1089/dia.2020.0169.

15. Pla B, Arranz A, Knott C, Sampedro M, Jiménez S, Hernando I, Marazuela M. Impact of COVID-19 Lockdown on Glycemic Control in Adults with Type 1 Diabetes Mellitus. J Endocr Soc. 2020;4:bvaa149. doi:10.1210/jendso/bvaa149.

16. Moreno-Domínguez Ó, González-Pérez de Villar N, Barquiel B, Hillman-Gadea N, Gaspar-Lafuente R, Arévalo-Gómez M, Herranz L. Factors Related to Improvement of Glycemic Control Among Adults with Type 1 Diabetes During Lockdown Due to COVID-19. Diabetes Technol Ther 2020. doi:10.1089/dia.2020.0550.

17. Di Dalmazi G, Maltoni G, Bongiorno C, Tucci L, Di Natale V, Moscatiello S, et al. Comparison of the effects of lockdown due to COVID-19 on glucose patterns among children, adolescents, and adults with type 1 diabetes: CGM study. BMJ Open Diabetes Res Care 2020. doi:10.1136/bmjdrc-2020-001664.

18. Tinti D, Savastio S, Grosso C, Donno V de, Trada M, Nugnes M, et al. Impact of lockdown during COVID-19 emergency on glucose metrics of children and adolescents with type 1 diabetes in Piedmont, Italy. Acta Diabetol 2021. doi:10.1007/s00592-021-01702-0.

19. Mesa A, Viñals C, Pueyo I, Roca D, Vidal M, Giménez M, Conget I. The impact of strict COVID-19 lockdown in Spain on glycemic profiles in patients with type 1 Diabetes prone to hypoglycemia using standalone continuous glucose monitoring. Diabetes Res Clin Pract. 2020;167:108354. doi:10.1016/j.diabres.2020.108354.

20. Predieri B, Leo F, Candia F, Lucaccioni L, Madeo SF, Pugliese M, et al. Glycemic Control Improvement in Italian Children and Adolescents With Type 1 Diabetes Followed Through Telemedicine During Lockdown Due to the COVID-19 Pandemic. Front Endocrinol (Lausanne). 2020;11:595735. doi:10.3389/fendo.2020.595735.

21. Capaldo B, Annuzzi G, Creanza A, Giglio C, Angelis R de, Lupoli R, et al. Blood Glucose Control During Lockdown for COVID-19: CGM Metrics in Italian Adults With Type 1 Diabetes. Diabetes Care. 2020;43:e88-e89. doi:10.2337/dc20-1127.

22. Bonora BM, Boscari F, Avogaro A, Bruttomesso D, Fadini GP. Glycaemic Control Among People with Type 1 Diabetes During Lockdown for the SARS-CoV-2 Outbreak in Italy. Diabetes Ther. 2020:1–11. doi:10.1007/s13300-020-00829-7.

23. Dover AR, Ritchie SA, McKnight JA, Strachan MWJ, Zammitt NN, Wake DJ, et al. Assessment of the effect of the COVID-19 lockdown on glycaemic control in people with type 1 diabetes using flash glucose monitoring. Diabet Med. 2021;38:e14374. doi:10.1111/dme.14374.

24. Caruso I, Di Molfetta S, Guarini F, Giordano F, Cignarelli A, Natalicchio A, et al. Reduction of hypoglycaemia, lifestyle modifications and psychological distress during lockdown following SARS-CoV-2 outbreak in type 1 diabetes. Diabetes Metab Res Rev. 2020:e3404. doi:10.1002/dmrr.3404.

25. Fernández E, Cortazar A, Bellido V. Impact of COVID-19 lockdown on glycemic control in patients with type 1 diabetes. Diabetes Res Clin Pract. 2020;166:108348. doi:10.1016/j.diabres.2020.108348.

26. Longo M, Caruso P, Petrizzo M, Castaldo F, Sarnataro A, Gicchino M, et al. Glycemic control in people with type 1 diabetes using a hybrid closed loop system and followed by telemedicine during the COVID-19 pandemic in Italy. Diabetes Res Clin Pract. 2020;169:108440. doi:10.1016/j.diabres.2020.108440.

27. Viñals C, Mesa A, Roca D, Vidal M, Pueyo I, Conget I, Giménez M. Management of glucose profile throughout strict COVID-19 lockdown by patients with type 1 diabetes prone to hypoglycaemia using sensor-augmented pump. Acta Diabetol. 2021;58:383–8. doi:10.1007/s00592-020-01625-2.

28. Cognigni M, D'Agostin M, Schiulaz I, Giangreco M, Carletti C, Faleschini E, et al. HbA1c and BMI after lockdown for COVID-19 in children and adolescents with type 1 diabetes mellitus. Acta Paediatr 2021. doi:10.1111/apa.15838.

29. Aragona M, Rodia C, Bertolotto A, Campi F, Coppelli A, Giannarelli R, et al. Type 1 diabetes and COVID-19: The "lockdown effect". Diabetes Res Clin Pract. 2020;170:108468. doi:10.1016/j.diabres.2020.108468.

30. Marigliano M, Maffeis C. Glycemic control of children and adolescents with type 1 diabetes improved after COVID-19 lockdown in Italy. Acta Diabetol 2021. doi:10.1007/s00592-020-01667-6.

31. Prabhu Navis J, Leelarathna L, Mubita W, Urwin A, Rutter MK, Schofield J, Thabit H. Impact of COVID-19 lockdown on flash and real-time glucose sensor users with type 1 diabetes in England. Acta Diabetol. 2021;58:231–7. doi:10.1007/s00592-020-01614-5.

32. Cotovad-Bellas L, Tejera-Pérez C, Prieto-Tenreiro A, Sánchez-Bao A, Bellido-Guerrero D. The challenge of diabetes home control in COVID-19 times: Proof is in the pudding. Diabetes Res Clin Pract. 2020;168:108379. doi:10.1016/j.diabres.2020.108379.

33. Christoforidis A, Kavoura E, Nemtsa A, Pappa K, Dimitriadou M. Coronavirus lockdown effect on type 1 diabetes management οn children wearing insulin pump equipped with continuous glucose monitoring system. Diabetes Res Clin Pract. 2020;166:108307. doi:10.1016/j.diabres.2020.108307.

34. Wu X, Luo S, Zheng X, Ding Y, Wang S, Ling P, et al. Glycemic control in children and teenagers with type 1 diabetes around lockdown for COVID-19: A continuous glucose monitoring-based observational study. J Diabetes Investig 2021. doi:10.1111/jdi.13519.

35. Brener A, Mazor-Aronovitch K, Rachmiel M, Levek N, Barash G, Pinhas-Hamiel O, et al. Lessons learned from the continuous glucose monitoring metrics in pediatric patients with type 1 diabetes under COVID-19 lockdown. Acta Diabetol. 2020;57:1511–7. doi:10.1007/s00592-020-01596-4.

36. Hosomi Y, Munekawa C, Hashimoto Y, Okamura T, Takahashi F, Kawano R, et al. Effect of COVID-19 pandemic on the lifestyle and glycemic control in patients with type 1 diabetes: A retrospective cohort study; 2020.

37. Al Agha AE, Alharbi RS, Almohammadi OA, Yousef SY, Sulimani AE, Alaama RA. Impact of COVID-19 lockdown on glycemic control in children and adolescents. Saudi Med J. 2021;42:44–8. doi:10.15537/smj.2021.1.25620.

38. Verma A, Rajput R, Verma S, Balania VKB, Jangra B. Impact of lockdown in COVID 19 on glycemic control in patients with type 1 Diabetes Mellitus. Diabetes Metab Syndr. 2020;14:1213–6. doi:10.1016/j.dsx.2020.07.016.

39. Önmez A, Gamsızkan Z, Özdemir Ş, Kesikbaş E, Gökosmanoğlu F, Torun S, Cinemre H. The effect of COVID-19 lockdown on glycemic control in patients with type 2 diabetes mellitus in Turkey. Diabetes Metab Syndr. 2020;14:1963–6. doi:10.1016/j.dsx.2020.10.007.

40. Karatas S, Yesim T, Beysel S. Impact of lockdown COVID-19 on metabolic control in type 2 diabetes mellitus and healthy people. Prim Care Diabetes 2021. doi:10.1016/j.pcd.2021.01.003.

41. Biancalana E, Parolini F, Mengozzi A, Solini A. Short-term impact of COVID-19 lockdown on metabolic control of patients with well-controlled type 2 diabetes: a single-centre observational study. Acta Diabetol 2020. doi:10.1007/s00592-020-01637-y.

42. Khare J, Jindal S. Observational study on Effect of Lock Down due to COVID 19 on glycemic control in patients with Diabetes: Experience from Central India. Diabetes Metab Syndr. 2020;14:1571–4. doi:10.1016/j.dsx.2020.08.012.

43. Falcetta P, Aragona M, Ciccarone A, Bertolotto A, Campi F, Coppelli A, et al. Impact of COVID-19 lockdown on glucose control of elderly people with type 2 diabetes in Italy. Diabetes Res Clin Pract. 2021:108750. doi:10.1016/j.diabres.2021.108750.

44. Sankar P, Ahmed WN, Mariam Koshy V, Jacob R, Sasidharan S. Effects of COVID-19 lockdown on type 2 diabetes, lifestyle and psychosocial health: A hospital-based cross-sectional survey from South India. Diabetes Metab Syndr. 2020;14:1815–9. doi:10.1016/j.dsx.2020.09.005.

45. Psoma O, Papachristoforou E, Kountouri A, Balampanis K, Stergiou A, Lambadiari V, et al. Effect of COVID-19-associated lockdown on the metabolic control of patients with type 2 diabetes. J Diabetes Complications. 2020;34:107756. doi:10.1016/j.jdiacomp.2020.107756.

46. Rastogi A, Hiteshi P, Bhansali A. Improved glycemic control amongst people with long-standing diabetes during COVID-19 lockdown: a prospective, observational, nested cohort study. Int J Diabetes Dev Ctries. 2020:1–6. doi:10.1007/s13410-020-00880-x.

47. Lippi G, Henry BM, Bovo C, Sanchis-Gomar F. Health risks and potential remedies during prolonged lockdowns for coronavirus disease 2019 (COVID-19). Diagnosis (Berl). 2020;7:85–90. doi:10.1515/dx-2020-0041.

48. Fonseca VA, Smith H, Kuhadiya N, Leger SM, Yau CL, Reynolds K, et al. Impact of a natural disaster on diabetes: exacerbation of disparities and long-term consequences. Diabetes Care. 2009;32:1632–8. doi:10.2337/dc09-0670.

49. Rubinstein A, Koffler M, Villa Y, Graff E. The Gulf War and diabetes mellitus. Diabet Med. 1993;10:774–6. doi:10.1111/j.1464-5491.1993.tb00163.x.

50. Ghosh A, Arora B, Gupta R, Anoop S, Misra A. Effects of nationwide lockdown during COVID-19 epidemic on lifestyle and other medical issues of patients with type 2 diabetes in north India. Diabetes Metab Syndr. 2020;14:917–20. doi:10.1016/j.dsx.2020.05.044.

51. Ruiz-Roso MB, Knott-Torcal C, Matilla-Escalante DC, Garcimartín A, Sampedro-Nuñez MA, Dávalos A, Marazuela M. COVID-19 Lockdown and Changes of the Dietary Pattern and Physical Activity Habits in a Cohort of Patients with Type 2 Diabetes Mellitus. Nutrients 2020. doi:10.3390/nu12082327.
